# The mental health implication of mpox: Enhancing care with genetic insights

**DOI:** 10.4102/jphia.v16i1.786

**Published:** 2025-01-24

**Authors:** Ovinuchi Ejiohuo

**Affiliations:** 1 Department of Psychiatric Genetics, Poznan University of Medical Sciences, Poznan, Poland; 2 Molecular and Cell Biology Unit, Poznan University of Medical Sciences, Poznan, Poland

**Keywords:** genetic testing, mpox, mental health, mental disorders, personalised medicine, precision psychiatry

## Abstract

The intersection of mpox and mental health is a critical concern, particularly for individuals with pre-existing mental disorders, who face heightened psychological stress and exacerbation of symptoms. This study explores the potential of genetic testing, such as Polygenic Risk Scores and pharmacogenetics, in enhancing mental disorders and mpox management. By tailoring treatment and prevention strategies to an individual’s genetic profile, clinicians can provide more personalised care, reducing adverse effects and improving outcomes. Furthermore, genetic insights can inform the development of safer vaccines and early interventions, particularly for vulnerable populations. The study underscores the importance of integrating mental and public health strategies, advocating for targeted research and fostering interdisciplinary collaboration to effectively address these complex health challenges.

## Introductory overview and possible solutions

The impact of mpox on individuals with pre-existing mental disorders is a critical area of concern because of the additional psychological stress and potential exacerbation of symptoms these patients might experience. mpox can intensify the mental health challenges faced by individuals with disorders such as depression, anxiety disorders, bipolar disorder and schizophrenia.^1,2,3^ This makes mpox a psychosocial stressor in itself, necessitating a holistic approach to care that addresses both the physical and psychological impacts of the disease.

Genetic testing offers significant potential in improving the treatment and management of both mental disorders and infectious diseases such as mpox. By leveraging techniques such as Polygenic Risk Scores (PRS), clinicians can gain valuable insights into an individual’s genetic predisposition to psychiatric conditions.^4,5,6^ This understanding allows for more personalised treatment plans, which are particularly crucial when dealing with a complex illness such as mpox. For example, knowing a patient’s genetic likelihood of adverse reactions to specific medications enables clinicians to choose treatments that minimise both psychological stress and physical side effects^7^ – an essential consideration for those already managing mental health issues. However, it is important to note that PRS may not pinpoint a specific disorder and is not a reliable predictor that an individual will develop the condition.^8^

Pharmacogenetics, which aligns treatments with an individual’s genetic profile, plays a key role in this personalised approach.^7,9^ In psychiatric care, this means reducing the trial-and-error process that often accompanies medication selection, leading to better outcomes and fewer side effects.^7^ When treating mpox in patients with mental disorders, such genetic insights become even more critical. Ensuring that antiviral treatments do not interact negatively with psychiatric medications protects the patient’s mental and physical well-being, making treatment more effective and safer.

Beyond treatment, genomic techniques also contribute to developing more effective and safer vaccines. Understanding the genetic factors that influence individual responses to vaccines allows developers to design protective immunisations that are less likely to cause adverse effects.^10^ This is particularly important for individuals with mental disorders, whose unique genetic profiles may affect their immune response. Tailored vaccines can prevent the onset of mpox while minimising the risk of exacerbating psychiatric symptoms, ensuring a comprehensive approach to patient care.

Early intervention represents a critical benefit of genetic testing. By pinpointing individuals at higher risk of severe mpox outcomes, we can initiate preventive measures earlier, thereby minimising the potential for the disease to exacerbate existing mental health conditions. This proactive approach not only addresses the physical symptoms of the disease but also mitigates the psychological strain, helping to prevent further decline in mental well-being. With this knowledge, we can develop personalised prevention strategies, such as tailored vaccination schedules or prophylactic treatments, enabling a more targeted and effective response to these complex health challenges.

In this interconnected framework, genetic testing is a crucial tool for integrating mental health and infectious disease management. By tailoring treatment and prevention strategies to an individual’s genetic profile, we can adopt a more comprehensive approach that addresses mental and physical health with greater precision and care.

## Recommendations and research areas to consider

Addressing the impact of mpox on mental well-being and mental disorders is crucial, particularly for vulnerable populations and underrepresented regions. Research and funding should prioritise the intersection of mpox and mental health, focusing on immunocompromised individuals, areas plagued by poverty, inadequate epidemiological and mental health surveillance, and regions affected by war and conflict. This intersection’s implications must be explicitly incorporated into mental and public health policy formulation and implementation. An integrated approach that combines mental health and public health strategies is essential to achieve the most effective outcomes.

Also, establishing coalitions and strategic partnerships is vital. Collaboration between clinicians (including psychiatrists and psychologists), public health experts, biomedical scientists, government agencies and relevant non-governmental organisations (NGOs) will be key to developing and implementing effective drug and vaccine production and ensuring robust regulatory frameworks and health response policies. This multidisciplinary approach will enhance the overall response to mpox, ensuring that physical and mental health impacts are addressed comprehensively and effectively.

## Conclusion

This viewpoint highlights the critical need for integrating genetic testing into the management of mpox, particularly for individuals with pre-existing mental disorders, to enable personalised treatment and prevention strategies that address both physical and mental health challenges, emphasising the importance of interdisciplinary collaboration and targeted research in vulnerable populations.
